# Bilateral Birdshot Retinochoroiditis and Retinal Astrocytoma

**DOI:** 10.1155/2017/6586157

**Published:** 2017-02-21

**Authors:** Sunil Mamtora, Yun Wong, Dugald Bell, Teresa Sandinha

**Affiliations:** Sunderland Eye Infirmary, Queen Alexandra Road, Sunderland SR2 9HP, UK

## Abstract

*Background*. This case highlights the importance of recognising multiple pathologies within the eye which may not necessarily be linked. Both birdshot retinochoroiditis and astrocytoma are rare conditions. The case underlines the need for early identification and treatment of birdshot retinochoroiditis with steroids and disease modifying drugs. Astrocytoma in the absence of tuberous sclerosis is also uncommon.* Case Presentation*. A 36-year-old male presented with 3-month history of bilateral progressive flashing lights and floaters. He was systemically well with no significant past medical history. Fundal examination revealed retinal vasculitis and active creamy lesions in the choroid radiating from the optic nerve. In the supranasal periphery of the right eye there was a raised white, jagged lesion protruding into the vitreous. Fluorescein angiogram and indocyanine green showed marked venous vasculitis, hypofluorescence, and disc leakage in keeping with birdshot retinochoroiditis. The supranasal lesion features were in keeping with astrocytoma and this was thought to be a coincidental finding.* Conclusions*. Retinal astrocytoma may be present as an isolated ocular finding; however, patients must still be investigated for tuberous sclerosis which is the most common association. Birdshot retinochoroiditis typically responds well to steroid therapy, and disease modifying drugs should be considered as soon as possible.

## 1. Background

This case highlights the importance of recognising multiple pathologies within the eye which may not necessarily be linked. Both Birdshot retinochoroiditis and astrocytoma are rare conditions requiring different treatments. The case underlines the need for early identification and treatment of birdshot retinochoroiditis with steroids and disease modifying drugs. Astrocytoma in the absence of tuberous sclerosis is also uncommon. This case and its clear pictures will help clinicians familiarise themselves with the clinical features of this rare condition.

## 2. Case Presentation

A 36-year-old male presented to the Sunderland Eye Infirmary (City Hospitals Sunderland) with a 5-week history of slowly progressing flashing lights and floaters in both eyes. His late presentation was only due to his subtle symptoms and he retained bilateral 6/9 vision. The patient had no significant past medical history or past ocular history and did not take any medication. He worked as an electrician and did not smoke or drink alcohol excessively. He had not had any recent foreign travel and regularly drove a car. There was no family history of any ocular or inflammatory conditions.

On examination visual acuity was 6/9 bilaterally and visual fields were full to confrontation. There was no RAPD and Ishihara plates were 17/17 bilaterally. Examination of the anterior segment revealed a few fine inferior keratic precipitates on both endothelium and one-plus anterior chamber inflammatory cells in the right eye, but a quiet left anterior chamber. The vitreous had one-plus inflammatory cells bilaterally; however the fundal view was clear with no vitreous haze.

Examination of the retina revealed bilateral peripheral flame shaped retinal haemorrhages with round creamy white chorioretinal lesions radiating out from the optic nerve. There was bilateral evidence of retinal venous sheathing in keeping with vasculitis. Both optic discs had blurred margins with fine haemorrhages in keeping with optic disc swelling with no evidence of spontaneous venous pulsation. In the right eye there was a supranasal retinal lesion which was craggy due to calcification, white, irregular, and jagged, protruding into the vitreous.

**Figure 1:**
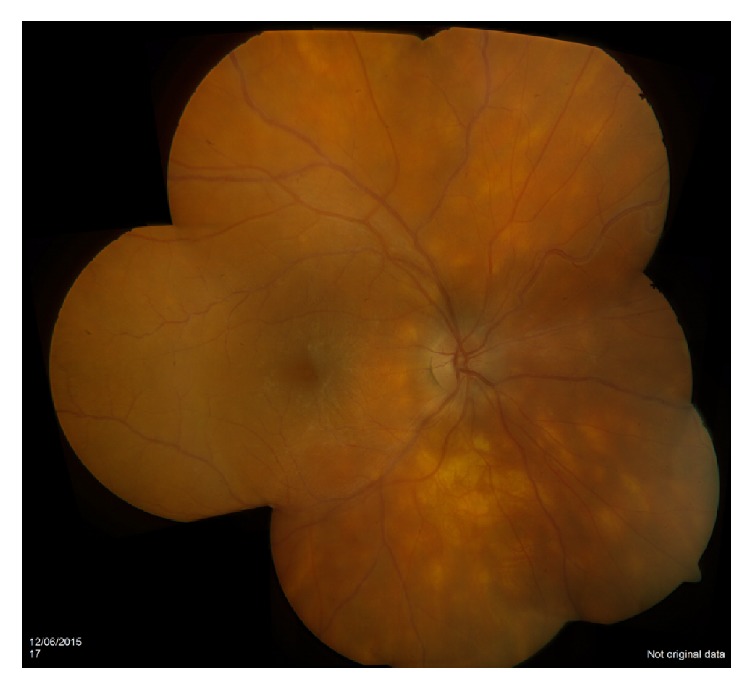
Colour fundus of the right eye revealing creamy retinal lesions in keeping with birdshot retinochoroiditis.

Due to the peripapillary creamy coloured lesions radiating from the optic disc bilaterally birdshot retinochoroiditis was highly suspected (Figure 1).

An inflammatory blood screen was completed on the day of presentation. These included FBC, U&E, CRP, ESR, LFTs, ACE, ANCA, RF, ANA, and a HLA A29. Blood was normal except for a raised rheumatoid factor 43.1 (normal range 0–14) and the patient was HLA A29 positive.

OCT completed on the day of presentation revealed the presence of a right epiretinal membrane; however there was no evidence of cystoid macular oedema in either eye.

**Figure 2:**
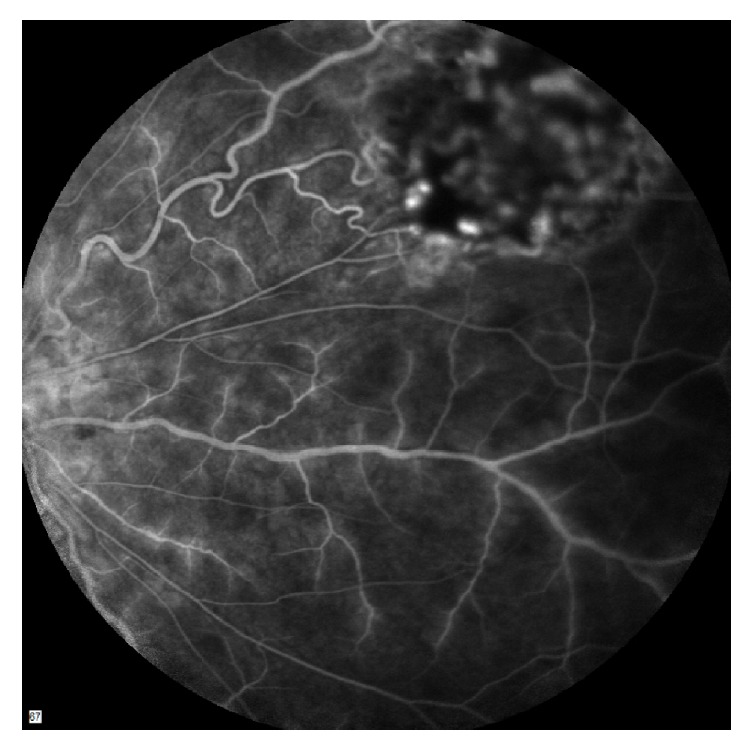
FFA of the right eye revealing retinal vasculitis and astrocytoma.

A fluorescein angiogram and indocyanine green were organised and completed two days after presentation. The FFA showed marked venous vasculitis in both eyes with leakage of dye due to breakdown of the inner blood retinal barrier and staining of the blood vessel wall with fluorescein (Figure 2).

The ICG showed multiple dark areas in keeping with hypoperfusion due to inflammation within the choroid. A chest X-ray was ordered to assess for features of sarcoidosis. This was reported as normal.

The multifocal and full field ERGs were normal in both eyes; however there was noted to be some asymmetry between the full field ERGs. The right eye showed evidence of lower amplitude responses in comparison to the left eye. This result was described as borderline statistically significant. This finding did not correlate with the intensity of vascular leakage on FFA.

**Figure 3:**
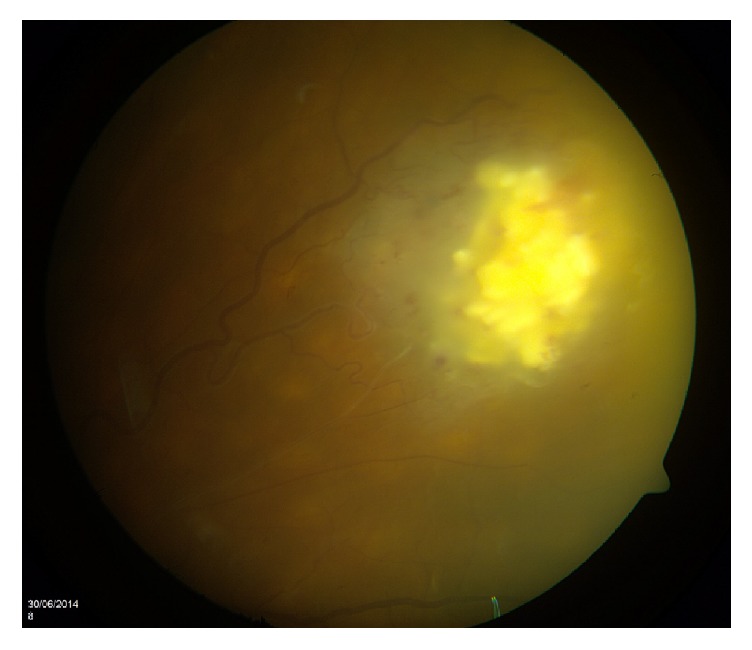
Colour fundus photo of the right eye revealing the white calcified astrocytoma and creamy retinal lesions in keeping with birdshot retinochoroiditis.

There was no change in the white retinal lesion on FFA or ICG and it appeared to have a calcified appearance (Figure 3). There was certainly no history of previous retinoblastoma. The clinical examination and investigation findings were in keeping with a diagnosis of retinal astrocytoma.

**Figure 4:**
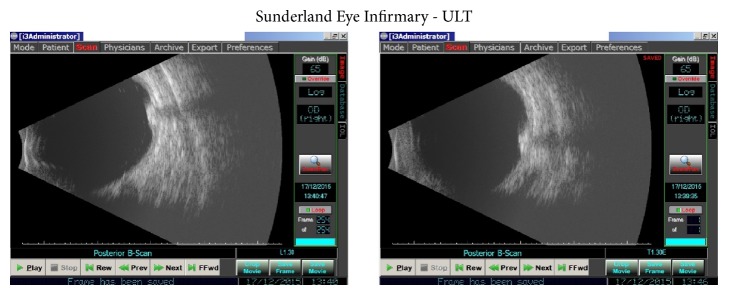
A B-scan report of the astrocytoma revealing calcification and high reflectivity. Impression: solid elevated mass SN equator. Patchy high reflectivity centrally persisting at low gain and with posterior shadowing in keeping with calcification. Lower reflectivity posterior and superior edges. No retinal detachment. PVD appears complete. Appearance in keeping with astrocytoma.

An ultrasound B-scan was also performed (Figure 4). This confirmed that the lesion was a solid elevated calcified mass, with high reflectivity and posterior shadowing. These features are in keeping with astrocytoma supporting the diagnosis.

With this diagnosis an MRI brain was completed due to astrocytomas close association with tuberous sclerosis. This was reported as normal with no brain lesions present.

The patient had a diagnosis of bilateral posterior and mild anterior uveitis. A differential diagnosis includes the white dot syndromes and specifically

- birdshot retinochoroiditis,
- multifocal choroiditis (MCP),
- sarcoidosis.

Due to the nature of the creamy choroidal lesions radiating from the disc and the positive HLA A29 the diagnosis of birdshot retinochoroiditis was made.

The unilateral white craggy retinal lesion protruding into the vitreous supranasal in the right eye had a differential diagnosis which included

- astrocytoma,
- amelanotic melanoma,
- old retinoblastoma.

As the lesion was asymptomatic and a raised white calcified mass which caused blockage of fluorescein on fundus fluorescein angiogram, a diagnosis of endophytic retinal astrocytoma was made.

The patient was commenced on 80 mg of oral prednisolone on the day of presentation. Mycophenolate mofetil (CellCept) was also started at a dose of 1 g BD with the steroid. The oral prednisolone was titrated down from 80 mg to 60 mg after one week with a subsequent weekly reduction by 10 mg to 20 mg. After 6 weeks of treatment the steroids were reduced by 2.5 mg per week with a plan to remain solely on CellCept if the eyes remained quiet.

In order to monitor the progress of this known chronic condition the patient's long-term follow-up plan included serial visual field assessments and electroretinography.

Alongside the steroid, bone and stomach prophylaxis was started in the form of lansoprazole and adcal D3. Astrocytomas routinely do not require treatment and the lesion will continue to be monitored to ensure that it does not increase in size or character.

The patient was reviewed one month after commencing high dose oral steroid and CellCept. At this visit he felt that the flashing lights and floaters had considerably improved and the vision was 6/6 bilaterally. Clinically there was less disc swelling and this was confirmed on FFA. The choroidal lesions also appeared less prominent and the anterior chambers and the vitreous were quiet bilaterally. Steroids were subsequently tapered down further; however the patient noticed a month later after he reduced his oral steroid dose from 20 mg to 15 mg that his vision had become a little hazy. He therefore increased his dose back up to 20 mg and attended clinic. At this clinic visit it was decided to titrate his oral prednisolone down by 2.5 mg per week from his 20 mg dose. The patient will continue his slow taper of oral steroid with the aim of maintaining him symptom free on CellCept.

During his follow-up and treatment of the birdshot retinochoroiditis the astrocytoma in the right eye did not change in nature. Due to the association between astrocytoma and tuberous sclerosis a routine MRI brain was organised which confirmed no intracranial lesions.

Both birdshot retinochoroiditis and retinal astrocytoma are rare conditions in themselves [1, 2] and have not been reported simultaneously. This case report provides clear images which highlight the conditions common features aiding diagnosis.

Retinal astrocytoma is a rare hamartoma which typically does not require treatment and is not detrimental to vision [3]. They are commonly a clinical diagnosis and appear as yellowish nodules which can develop into larger mulberry type lesions which autofluoresce.

Astrocytomas are typically static lesions which may become calcified over time and on FFA they may have a prominent permeable superficial vascular network which can result in hyperfluorescence [4]. Indocyanine green more often reveals hypocyanescene, most prominently during the late phases. Histologically they show fibrillary astrocytes with small oval nuclei. Most are endophytic, protruding into the vitreous; however exophytic subretinal astrocytomas can occur. Retinal astrocytomas may occasionally occur as incidental solitary lesions such as in our patient; however they are most commonly seen in patients with tuberous sclerosis [5] and less commonly in neurofibromatosis 1 and retinitis pigmentosa. 50% of tuberous sclerosis patients have retinal astrocytomas which may be multiple and bilateral.

Due to the heavy association with tuberous sclerosis, we fully investigated our patient for the condition. This autosomal dominant phacomatosis characterised by the development of hamartomas in multiple organs classically presents with a triad of epilepsy, mental retardation, and adenoma sebaceum [6]. Around 60% of patients may develop the condition sporadically [3], and therefore asymptomatic patients should be fully investigated including an MRI brain due to the risk of intracranial paraventricular subependymal astrocytic nodules and giant astrocytic hamartomas [7].

Birdshot retinochoroiditis is a posterior uveitis which is typically bilateral and results in inflammation of the choroid and the retina [3]. It commonly presents in the 3rd or 6th decade and affects women more commonly than men. It is associated with HLA A29 [8], and patients present with floaters and visual disturbance. On examination the choroid shows evidence of small creamy spots radiating out from the optic nerve like birdshot from a shotgun. Fluorescein angiograms show staining at the disc and hyperfluorescence due to leakage [9]. Indocyanine green identifies the choroid and commonly reveals well-defined oval hypofluorescent spots [10]. In acute birdshot retinochoroiditis the electroretinogram may develop a reduced B wave (electronegative) which, if treated, may well reverse over a period of time [11]. It typically responds well to high dose systemic steroids which can be tapered but optimal treatment involves the use of disease modifying drugs such as mycophenolate mofetil (CellCept) [12].

This case highlights some typical presenting features of birdshot retinochoroiditis underlining its association with HLA A29 as well as a few atypical features such as fine inferior keratic precipitates and flame shaped haemorrhages. It also emphasises the need to appreciate the fact that separate rare pathologies can exist within the eye.

## 3. Conclusions

When two apparently distinct lesions are detected in the eye, it is important to consider whether they are part of the same disease process (Occam's Razor) or two simultaneously presenting but separate and unconnected pathologies (Hickam's Dictum). The latter applies in this case.

Retinal astrocytoma may be present as an isolated ocular finding; however patients must still be investigated for tuberous sclerosis which is the most common association. Birdshot retinochoroiditis typically responds well to steroid therapy and disease modifying drugs should be considered as soon as possible.
